# Open Access publishing: the continuing development of the *Journal of Nutritional Science*

**DOI:** 10.1017/jns.2017.72

**Published:** 2018-01-12

**Authors:** Paul Trayhurn

**Affiliations:** Editor-in-Chief of the *Journal of Nutritional Science*, Obesity Biology Unit, University of Liverpool, Liverpool L69 3GA, UK and Clore Laboratory, University of Buckingham, Buckingham MK18 1EG, UK

The *Journal of Nutritional Science (JNS)* was established in the early part of this decade as an initiative between the Nutrition Society and Cambridge University Press^(^^1^^)^, with the first volume appearing in 2012. The founding Editor-in-Chief, Professor Philip Calder, was at the time also Editor-in-Chief of the *British Journal of Nutrition (BJN)*. This reflected the original concept that the *JNS* would be closely allied to the *BJN*, with the same Editor-in-Chief and a common Editorial Board. The new journal was in part intended to provide a vehicle for articles that while scientifically sound were not able to be published by the *BJN*, because of the pressures on space in the Society's flagship journal. A close association between the *JNS* and the *BJN* continued when Professor Graham Burdge took over as Editor-in-Chief of both journals in 2013.

Increasingly, the *JNS* has been receiving direct submissions and these now form most of the material published rather than articles originally submitted to the *BJN* (although these continue as part of the content). This evolution underlies the growing sense that the ‘new’ journal should forge its own identity and be clearly distinct from its esteemed parent. As part of this transition, it has been agreed that the *JNS* should have its own Editor-in-Chief, separate from that of the *BJN*, and I am delighted to say that I assumed this role in October 2017. I am most grateful to my predecessor, Professor Burdge, for all his work on the journal, and I am very pleased that he has agreed to continue to serve the *JNS* as Deputy Editor. Over the next year, the *JNS* will also be establishing its own Editorial Board, though I anticipate that there will be some overlap with the *BJN*. I would like to thank those who have agreed to continue on the *JNS* Board in the interim.

The defining characteristic of the *JNS* is, of course, that it is a fully Open Access (OA) journal. OA publishing has expanded dramatically since the concept began to gain traction some 15 years ago, and there are now both fully OA and hybrid models. OA as a route for the dissemination of scientific information transfers the cost of publication from the consumer to the producer, with subscription charges being replaced by fees paid by authors; the key advantage is that there is no paywall barrier to the availability of what is published with the result that the reach of scientific papers is maximised – and potentially the number of citations^(^^2^^)^. There is now a range of highly successful OA journals, amongst the best known of which are *PLOS ONE* and the *Frontiers* series. Fully OA journals are proliferating at a rapid rate, and many will be familiar with the daily influx of email requests to submit an article to a new OA journal, often from an obscure publisher. Some of these journals have been termed ‘predatory’, and until recently Jeffrey Beall of the University of Colorado-Denver maintained an extensive blacklist of such journals. Among the dangers of ‘predatory journals’ is that they risk undermining the concept of OA publishing. A number of new OA journals, of course, have impeccable credentials, being published under the auspices of the Royal Society in the case of *Open Biology*, for example.

The *JNS* has the strengths and combined authority of the Nutrition Society and Cambridge University Press behind it, the Press being the world's oldest publishing house, having had ‘letters patent’ granted by King Henry VIII of England in 1534. OA journals established, owned and run by learned societies are in practice a return to the origins of scientific publishing. The majority of long-established, discipline-based journals were set up by learned societies, these including, for example, the *BJN*, the *Journal of Physiology* and the *Biochemical Journal* in the UK, and the *American Journal of Physiology* and the *Journal of Nutrition* in the USA *–* as well as the world's earliest and longest-running scientific journal, the Royal Society's *Philosophical Transactions*.

Academic journals owned and run by commercial publishers are a relatively recent development, and it has now been argued that learned societies should exercise their moral authority and reclaim the guardianship of publishing^(^^3^^)^. Indeed, in 1957, David Martin, the then Assistant Secretary of the Royal Society, stated that ‘Scientific Societies should be the guardians of the quality of scientific publication of original work in learned journals’^(^^3^^)^. Given the development of OA and of predatory journals, this is especially apposite – and with the *JNS*, taking guardianship is exactly what the Nutrition Society is doing.

The *JNS*, which is now in its seventh year, will continue to evolve. I am concerned, as are Editors of every journal, to ensure that the review process is as speedy as possible, consistent with the maintenance of high standards. The remit covers the full spectrum of the nutritional sciences – from genetic, molecular and cellular studies to metabolism and whole-body physiology, through to dietary surveys, epidemiology and public health issues. Indeed, the journal takes a catholic view of what constitutes nutrition, and this includes studies using cell culture systems and plant extracts when these have nutritional relevance^(^^4^^)^. A wide range of article types are welcomed: Primary Research Papers, Brief Reports, Reviews, Perspectives articles, Systematic Reviews, Workshop Reports, Letters to the Editor and Obituaries. We are pleased that the *JNS* is listed in key indexing and abstracting services, including PubMed, PubMed Central, Scopus, Essential Sources Citation Index and the Web of Science.

The goal for the *JNS* is to become a leading nutrition journal and to be the vehicle of choice for those wanting full OA publication. We look forward to a successful future and to receiving your high-quality manuscripts.
